# Use of Information and Communications Technology in Family Services and Its Perceived Benefits amid the COVID-19 Pandemic in Hong Kong—Jockey Club SMART Family-Link Project

**DOI:** 10.3390/ijerph192315634

**Published:** 2022-11-24

**Authors:** Hilary Pui-Yee Ho, Agnes Yuen-Kwan Lai, Ricky Yu-Kwong Kwok, Tai-On Kwok, Edward Chow, Teresa Yip, Wai-Yan Tang, Kam-Wing Leung, Eliza Lam, Kam-Yin Lam, Terry Shuk-Yin Tsui, Wai-Fong Lok, Pui-Wah Yam, Shuk-Fong Wong, Chiu-Man Wong, Kerin Kwok-Wing Cham, Bonny Yee-Man Wong, Sai-Yin Ho, Man-Ping Wang, Tai-Hing Lam

**Affiliations:** 1 School of Public Health, The University of Hong Kong, Hong Kong; 2 School of Nursing, The University of Hong Kong, Hong Kong; 3 Department of Electrical and Electronic Engineering, The University of Hong Kong, Hong Kong; 4 Hong Kong Metropolitan University, Hong Kong; 5 Technology-Enriched Learning Initiative, The University of Hong Kong, Hong Kong; 6 The Hong Kong Catholic Marriage Advisory Council, Hong Kong; 7 Hong Kong Sheng Kung Hui Welfare Council Limited, Hong Kong; 8 Hong Kong Christian Service, Hong Kong; 9 Caritas-Hong Kong, Hong Kong; 10 Tung Wah Group of Hospitals, Hong Kong; 11 Hong Kong Children & Youth Services, Hong Kong; 12 Hong Kong Family Welfare Society, Hong Kong; 13 The Neighbourhood Advice-Action Council, Hong Kong; 14 Christian Family Service Centre, Hong Kong; 15 St. James’ Settlement, Hong Kong; 16 International Social Service Hong Kong Branch, Hong Kong; 17 School of Nursing and Health Studies, Hong Kong Metropolitan University, Hong Kong

**Keywords:** information and communications technology, COVID-19, family services, pandemic, perceived benefit, Hong Kong

## Abstract

Introduction: We examined information and communications technology (ICT) use in family services and its perceived benefits and barriers amid the COVID-19 pandemic in Hong Kong. Methods: We invited all family service social workers of 12 NGOs to complete an anonymous online questionnaire in May 2020 (Part A) and a management representative from each NGO to provide written feedback on ICT use in January 2021 (Part B). Results: In Part A, of 255 respondents (response rate: 67.3%), perceived ICT use in conducting programmes, groups, casework and preventive family programmes showed net increases of 33.9–54.5%; perceived ICT use in different work processes showed net increases of 18.8–48.6%. The three most common perceived benefits were “providing another option to service users” (74.2%), “more convenient service” (60.2%) and “maintaining normal service” (59.7%). In Part B, 12 management representatives consistently reported positive impacts of ICT use on family services. They shared actionable strategies (e.g., ICT skill-related training) and concerns about data privacy and management. Conclusions: ICT use in family services markedly increased amid the COVID-19 pandemic in Hong Kong. It helped make the services more accessible especially when the pandemic restricted face-to-face services.

## 1. Introduction

Information and communications technology (ICT) is essential to social work services. ICT enables workers to cope with the unprecedented coronavirus disease (COVID-19) pandemic, maintain communication with clients and provide essential services. 

Since the first confirmed COVID-19 case in late January 2020, voluntary mask-wearing in public has been nearly universal in Hong Kong, which became mandatory in July 2020 [1]. Other control measures included social distancing (e.g., work-from-home, school suspension, restricted operations in catering and entertainment venues), small district-based COVID-19 testing, border closure/control and quarantine. The severe economic downturn and rising unemployment raised stress in families, especially those deprived. Such stress, poor living conditions and tensions among family members were detrimental. Demands for counselling services on mental health and family relationships surged [2,3]. Integrated family service is one of the major streams of social services, and family service social workers had to overcome such unprecedented challenges to maintain services and provide urgent assistance. 

In Hong Kong, 67 Integrated Family Service Centres (IFSCs) and Integrated Service Centres (ISCs) provide comprehensive family services. IFSCs/ISCs aim to promote resilience and handle various problems through individual sessions, groups and programmes on life education, family relationship and holistic health enhancement, counselling and referral service [4]. Table 1 shows the complexity of service needs, service targets, format and examples of four family service components, including “Casework”, “Groups”, “Programmes”, and “Preventive family programmes” [5]. The intensity of intervention depended on the complexity of service needs. 

Before the COVID-19 pandemic, most family services were delivered face-to-face instead of online using ICT. The reliance on conventional face-to-face over online service could be explained on three levels. At the system level, financial, infrastructure, and technical constraints have limited ICT use [6]. At the service level, online service may seem less appropriate for complex cases requiring intensive counselling and immediate tangible support. At the individual level, social workers are concerned about risks in handling personal data and the limitations of delivering services online [7,8].

We searched PubMed and Social Work Abstract databases, including peer-reviewed journals from a wide range of professions covering medicine and social sciences with keywords including ‘ICT’, ‘pandemic’ and ‘social work’, up to 24 July 2022 and found four entries. Three were qualitative studies, including one with 23 social workers that explored their experiences of ICT use in practice and influencing factors [9]. Another study with 27 social workers and 22 service users explored their views on ICT-related service delivery [10]. The third qualitative study with two social workers and nine service users explored the importance of digital communicative competencies in handling and accessing information [11]. The fourth was our sister study on the internet-based training, learning and sharing platform (i-TLS) use of 313 social workers with quantitative and qualitative evaluation [12]. No reports have evaluated ICT use in community social services, including family services.

In 2018, the Hong Kong Jockey Club Charities Trust initiated and funded a 4-year SMART Family-Link (JCSFL) Project to advance family services by leveraging ICT. The Project is a collaboration of the School of Public Health (SPH) and Teaching-Enriched Learning Initiatives (TELI) of the University of Hong Kong (HKU) and all 26 Integrated Family Service Centres (IFSCs)/Integrated Service Centres (ISCs) operated by 12 NGOs. Details of the Project (https://www.jcsmartfamilylink.hk (accessed on 29 September 2022)) have been reported elsewhere [13]. The Project aims to benefit more family service users and enhance the quality by leveraging ICT. The expected positive changes (benefits) include (i) providing another (online) service option, (ii) more convenient services, (iii) maintaining normal service during the pandemic, and (iv) broadening service coverage with increased varieties and formats of activities, (v) reaching the unreachable who cannot attend or reject face-to-face service, (vi) improving service efficiency, (vii) early identification of high-risk groups, and (viii) destigmatisation (service users will not be stigmatised when seeking help through ICT use on sensitive issues, such as family violence).

Grounded on the co-work model of co-learning, co-creation and co-sharing between NGO partners and academia, the Project had five strategic themes: SMART living habits (e.g., hand washing and masking); SMART Emotion (e.g., encouraging positive emotion); SMART Coping (e.g., ways to enhance positive thinking); SMART communication (e.g., positive family communication); and SMART parenting (e.g., positive reinforcement to children); and co-created 12 digital family games [13], 242 picture and animated e-messages and 15 mobile game applications. They were used to promote and implement family service and public education activities. Figure S1 shows examples of picture and animated e-messages on five strategic themes of family well-being and the 15 mobile game applications on three strategic themes. 

The Project also co-created two ICT platforms, E-message Portal and Evaluation Tool Builders. Figure S2 shows screenshots of the two platforms. The E-message Portal is a web-based platform comprising a simple clip-art library and word editing functions. The picture e-messages produced can be mass disseminated to service users via WhatsApp and Short Message Service (Figure S2). The Evaluation Tool Builder helps social workers build electronic forms or questionnaires for activity recruitment, evaluation and follow-up. Tables S1 and S2 show the applications of co-created ICT tools and platforms in 4 family service components (“Casework”, “Groups”, “Programmes”, and “Preventive family programmes”) and six work processes. The six work processes included planning (designing and preparing the activities), promotion (publicising the activities), recruitment (recruiting service users), implementation (conducting the activities), evaluation (collecting feedback from service users) and follow-up (follow-up service users after the activities).

The objectives of this study were (i) to examine the changes in perceived ICT use in family service components and work processes, (ii) to explore the associations of the family service components and work processes with increased ICT use and perceived benefits, and (iii) to obtain NGO’s lessons learnt from COVID-19 and in ICT use, the perceived barriers and preference in ICT use, amid COVID-19 pandemic. We hypothesised significant associations of perceived benefits with the number of family service and work process components with a perceived increase in ICT use amid the COVID-19 pandemic. The significance of this study was to better understand the ICT use in different family service and work process components and the barriers that might help the NGO management level for better resource allocation and formulate key actionable strategies for service improvement.

## 2. Materials and Methods

### 2.1. Sampling and Procedures

This cross-sectional study involved both quantitative (Part A) and qualitative components (Part B). In Part A, an anonymous, self-administered online questionnaire was sent to all 410 social workers of 26 IFSCs/ISCs from 12 NGOs in May 2020. Emails were sent to each participating NGO to encourage their staff to answer the online survey within two weeks of receiving the link to the online questionnaire. In Part B, we obtained qualitative feedback on seven open-ended questions from 12 NGO management representatives in January 2021. Owing to the drastically increased workload of social workers during the COVID-19 pandemic, although it would be a limitation in the interpretation, we invited one representative from each NGO to respond on behalf of their centres. The study was approved by the Institutional Review Board of the University of Hong Kong/Hospital Authority Hong Kong West Cluster (UW 20-385).

### 2.2. Measures

#### 2.2.1. Part A: Quantitative Online Survey

As no validated questionnaires on ICT use and benefits in family services were found, outcome-based questions were developed and used to assess ICT use in family services, work processes, and perceived benefits.

Perceived ICT use in family services: Four questions were used to assess the ICT use in family service components; we asked, “Did ICT use change during the COVID-19 pandemic (February to April 2020) compared with before the pandemic (December 2019 to January 2020)?” on each component. Response options were “Decreased”, “No change”, “Increased”, and “Not applicable/Did not use”. The net change in perceived ICT use was calculated by subtracting the percentage decreased from the percentage increased. The response options were also recoded to indicate if an increase in perceived ICT use occurred (“Yes” for choosing “Increased” and “No” for choosing other options). The number of service components with a perceived increase in ICT use was summed (range 0 to 4).

Perceived ICT use in work processes: Two questions were used to assess ICT use in the six work processes: (Q1) “Before the COVID-19 pandemic (December 2019 to January 2020), in what work processes have you used ICT?”; (Q2) “During the COVID-19 pandemic (February to April 2020), in what work processes have you increased ICT use?”. Response options for both questions included the six work processes and “Others”. Because the work processes of promotion, recruitment, implementation, and evaluation were core functions and with the most perceived increase in ICT use, the number of these four core work processes with a perceived increase in ICT use was summed (range 0–4). Respondents were deemed to have any perceived ICT use before the pandemic or any increased perceived ICT use during the pandemic if one or more specific work processes were selected in Q1 and Q2, respectively. 

Perceived benefits of using ICT: One question was used to explore the perceived benefits of using ICT. We asked, “What benefits have increased ICT use brought you?” Based on the project objectives, response options included “Provided another service option”, “Improved service convenience”, “Maintained normal service”, “Broadened service coverage”, “Reached the unreachable”, “Increased service efficiency”, “Early identified high-risk groups”, “Destigmatisation”, “Others” and “None”. Respondents could choose one or more options. Each perceived benefit was analysed as “yes” vs. “no”. 

#### 2.2.2. Part B: Qualitative Feedback

A qualitative survey with seven open-ended questions was sent via email to management representatives (centre supervisors or heads of family service) of the 12 NGOs in January 2021. All 12 NGOs (100%) returned their answers. With the aim of service improvement, the seven open-ended questions focusing on service needs, experience, lessons learnt, perceived barriers and preference in ICT use included: (i) “How does COVID-19 affect the family service in your organisation?”; (ii) “How ICT use helped overcome difficulties caused by the COVID-19 pandemic to family services?; (iii) “What was the most important achievement of family services through ICT use during the COVID-19 pandemic?”; (iv) “What is the most important lesson learnt from the COVID-19 pandemic and ICT use for the organisation?”; (v) “What is your vision of ICT use in family service in the future?”; (vi) “Do you expect barriers and problems of ICT use in the future?” and (vii) “What are the most important areas (such as barriers and facilitators) in ICT use in family services?”.

### 2.3. Statistical Analyses

All quantitative analyses were conducted using IBM SPSS version 27 (Armonk, NY, USA). Descriptive statistics were used to describe the respondents’ characteristics, ICT use and perceived benefits. Respondents’ characteristics included sex, age group (18–29, 30–39, 40–49, 50–59 years), education (undergraduate, postgraduate) and rank (supervisor, frontline social worker), which were considered covariates. 

Perceived benefits in using ICT (“Yes” versus “No”) were the dependent variables. The independent variables were the number of family service components and work processes. Logistic regression was used to explore the associations of the perceived benefits with (i) the number of main components of family services with increased ICT use and (ii) the number of work processes with increased ICT use, with adjustment of covariates (sex, age, education and rank). 

Since the qualitative survey was completed in written format, no transcription was required. Framework analysis was employed with an organised structure of themes [14]. The themes included service needs, social workers’ experience, lessons learnt, and perceived barriers and preferences in ICT use. These were identified by the two authors, a social worker (HPYH) and an experienced researcher (AYKL). All codes and themes and their summary were discussed and reviewed among Principal/Co-Investigators (THL and SYH) to ensure consistency and reliability of codes and themes identified in the qualitative analysis process.

Different strategies were used to enhance the research vigour. Member checking was conducted by asking respondents to give feedback on emerging interpretations to ensure a good representation of their realities. Two researchers analysed each feedback. Peer debriefing was held to review the consistency of identified information with other co-investigators. Triangulating qualitative findings with the quantitative results were performed to maximise the validity of the findings.

## 3. Results

### 3.1. Quantitative Online Survey

Among the 410 social workers invited, 286 (70%) responded. After further examination, 31 who did not use ICT were excluded, leaving 255 for analysis. Table 2 shows that of 255 respondents, most were women (73.2%), aged 30–49 (65.9%), had postgraduate education (54.0%) and were frontline social workers (89.4%).

Table 3 shows respondents reported a perceived increase in ICT use in all four family service components (from 40.6% for preventive family programmes to 60.4% for groups). At the same time, few had decreased ICT use (from 3.1% for casework to 6.7% for programmes and preventive family programmes) during the COVID-19 pandemic. All four service components showed positive net changes (percentage increased minus percentage decreased) in perceived ICT use, from 33.9% for preventive family service to 54.5% for groups.

Table 4 shows that perceived ICT use in various work processes before the COVID-19 pandemic ranged from 6.3% for others to 40.0% for promotion. The prevalence of perceived increase in ICT use was higher in work processes of activities promotion (48.6%), implementation (46.3%), recruitment (38.4%) and evaluation (36.9%), but low in activities planning (18.8%) and follow-up (15.5%). 

Table 5 shows that the most common perceived benefits of ICT use during the COVID-19 pandemic were “Provided another service option” (74.2%), followed by “Improved service convenience” (60.2%), and “Maintained normal service” (59.7%), broadened service coverage (41.6%), reached the unreachable (26.2%) and improved service efficiency (24.9%). 

Each perceived benefit was analysed as a dependent variable. Regarding the number of family service components with a perceived increase in ICT use (analysed as an independent continuous variable, range 0–4), it showed a significant association with the perceived benefits of broadening service coverage [adjusted odds ratio, (95% confidence interval), 1.63, 1.23–2.15], providing another service option to users (1.38, 1.00–1.90), and improving service efficiency (1.38, 1.02–1.87). Regarding the number of core work processes with a perceived increase in ICT use (analysed as an independent continuous variable, range 0–4), it showed significant association with the perceived benefits of broadening service coverage [adjusted odds ratio (95% confidence interval), 1.41, 1.14–1.74], reaching the unreachable (1.39, 1.08–1.78), and maintaining normal service (1.24, 1.01–1.53).

### 3.2. Qualitative Feedback

#### 3.2.1. Theme One: Increased Service Needs and ICT Use during COVID-19

The demand for service was increasing dramatically. Respondents were striking a balance between providing service and maintaining infection control measures (such as social distancing). Tangible service requests soared in casework service, particularly in financial assistance applications, accommodation arrangements and settling urgent family issues.

“*During the pandemic……It caused increasing conflict among family members, inducing mental health problems, and raised financial / accommodation problems……almost double caseloads in family service, the advice of “Keeping Social Distance” made social workers more difficult to conduct a home visits and deliver face-to-face counselling, group or programme…… Social workers should strike a balance between providing service and safety measure(s).*” (IFSC C)

“*Instead of family relationship issues, nearly 90% of service users requested tangible support. All social workers were preoccupied with the funding and resource applications while we were still responding to those risky families.*” (IFSC B)

Most services were transformed from face-to-face to hybrid mode and formulated a new normal in service delivery. Family social workers and service users needed to acquire ICT skills to maintain and receive the service. The social distancing measures and the fear of infection in service users led to great challenges, especially for those admitted to the hospital. It might affect the service quality and client-worker relationship.

“*We have conducted most of the intakes/casework services via telephone or Zoom with clients and social workers sitting in different rooms……Our colleagues still render timely crisis interventions, such as outreach, home visits and escorting clients to clinics/hospitals. However, once clients have been admitted into hospitals, caseworkers are usually not allowed to visit them, which makes the case assessment, social investigation and formulation of welfare plans more indirect or difficult.*” (IFSC E)

“*The activities were suspended in the centres. The centres switched to online enrolment and online activities in May 2020. In 2020, more than 70% of the total activities of the centres were held online.*” (IFSC H)

“*Using social media, enhancing intervention skills in an online programme, and using ICT tools were all new aspects and techniques different from traditional social work training. To adapt to the “new normal”, more evidence-based research about using ICT in social work intervention should be enhanced.*” (IFSC I)

#### 3.2.2. Theme Two: Facilities and Support Provided by JCSFL Met the Demand for ICT-Related Services during the Pandemic

Both hardware (e.g., equipment and ICT infrastructure) and software (e.g., i-Action training sessions and self-paced i-TLS online co-learning and sharing platform) from the JCSFL helped social workers to transform face-to-face mode into a hybrid mode to maintain service continuation and meet the high demand of ICT knowledge and skills from the pandemic crisis despite the limitation from social distancing and work-from-home practice. Social workers adopted a new work model with good adaption of ICT use.

“*Our team is more prepared with the equipment, training classes, and online co-learning and sharing platform. We were more adaptive to using ICT now, although some teammates were reluctant before the pandemic. For example, teammates are accustomed to using ICT to attend team meetings or training now.*” (IFSC A)

“*ICT programmes, like Zoom, YouTube videos, games from i-Action, etc., serve as a good means for facilitating online interactions between social workers and service users.*” (IFSC E)

“*Online broadcasts, Zoom programmes/ groups, and even webinars were used last year. It bridged up using virtual means when face-to-face programmes were suspended. More people who worked from home or the general working-class participated in our online programmes without geographical restrictions, which enlarged our service coverage…….we had organised 45 online programmes/groups for almost 550 participants from March to December 2020 that received very encouraging feedback.*” (IFSC L)

ICT provided flexible and effective options to supplement the conventional face-to-face procedures in different service and work process components with unexpectedly positive results.

“*At the casework level, social workers can use FaceTime, WhatsApp, WeChat or any online communication applications to replace the face-to-face interview. Service users can also provide their information via WhatsApp for funding or tangible service applications. At the group and programme levels, service users can register programmes via Google form using links and QR codes on Facebook. At the implementation level, social workers use online tools, e.g., YouTube, Zoom, Facebook or WhatsApp, to maintain support groups and programmes…participants may encounter some problems or technical issues. They might ask our social workers through WhatsApp or share their experience in the WhatsApp group with other participants. At the public information delivery level… we have increased the use of social media… i.e., the quarter leaflet or IFSC will be posted on Facebook each quarter to ensure our service users can keep receiving the information of IFSC.*” (IFSC H)

#### 3.2.3. Theme Three: Experience and Lesson Learnt from COVID-19 and in ICT Use

After using ICT, the organisations were more responsive to change and adjusting to the new normal amid COVID-19. It provided service users with more interesting and attractive groups/programmes, which could accommodate some special target groups, caregivers and single parents, and overcome the time and space constraints.

“*We are more flexible and responsive to change and learn the new normal.*” (IFSC A)

“*There was an adjustment period for the use of ICT tools such as Zoom or other streaming applications for both the workers and the service users.*” (ISC D)

“*ICT is very valuable during COVID-19 as it helps reconnect people with little interactions with others or even have to observe social distancing rules…. In social welfare services, we value a lot of human interactions and may not be very familiarised with ICT. So, COVID-19 unexpectedly provides us with a good learning opportunity and experience to equip ourselves with ICT.*” (IFSC E)

“*Online activities could not only attract new service users but also break the territorial and time restrictions, allowing service users flexibility to participate in the programme anywhere and at any time.*” (IFSC H)

“…. *Online approach was suitable for special target groups such as single parents and caregivers. ICT helped them gain access to our services despite their limited time. The adaptation and acceptance of the service users in receiving online services were unexpectedly high. … interpersonal connection is still important….which was hard to achieve online. Face-to-face counselling service is irreplaceable.*” (IFSC I)

#### 3.2.4. Theme Four: Perceived Barriers to ICT Use

Most service users were from financially deprived families and the elderly. Such disadvantaged service groups might lack equipment and have low ICT literacy to fit into the online service mode. Besides, the limited private space might hinder service users’ motivation to use ICT at home.

“*Most of our service targets are grassroots families with difficulties buying ICT equipment and paying for the internet. They are not familiarised with the related technologies as well. It hinders their motivation and is a barrier to ICT use.*” (IFSC E)

“*Due to the small living environment, it was difficult for them to have a private space for online programmes or learning. The unstable Wi-Fi system or low speed is another barrier, especially for grassroots families and the elderly.*” (IFSC F)

“…*confidentiality of personal and organisational data would be another issue to be concerned about using ICT in the family service. During the intervention process with ICT use, it would be difficult for us to ensure that the content of intervention would not be recorded or spread out.*” (ISC K)

Some social workers stated they needed more time to be ready to use ICT in delivering their services because of their low confidence in acquiring new ICT skills and their use of traditional working styles. Workers also highlighted the importance of intensive face-to-face counselling contact for managing crisis cases that facilitated rapport building between service users and counsellors and should not be replaced by ICT.

“*In facing the rapid development of ICT, it would be difficult for social workers to catch up with its ever-changing advancement.*” (IFSC G)

“*Some social workers were reluctant to change because of their traditional working styles and heavy IFSC workload, which hindered the ICT development in organisations.*” (IFSC B)

“*The ICT service model might not be feasible for intensive counselling in some cases requiring more in-depth interactive contact.*” (IFSC A)

“…*through online counselling, rapport building between service users and counsellors would not be easy; some service users might prefer to come to the social service centre physically.*” (IFSC I)

#### 3.2.5. Theme Five: Preference for ICT Use

In addition, data security and clients’ privacy were major concerns when using ICT in service. Our respondents suggested conducting more professional training to enrich staff’s ICT literacy, providing more actionable guidance on ethical issues and data management, deliver more health-related training related to COVID-19 and mental health management to improve work efficiency and service outcomes. IFSCs/ISCs lent those who attended their groups/programmes ICT devices and provided the necessary equipment for some users who did not possess these related to attending online activities.

“*Owing to a dramatic increase in the demand for mental health enhancement service during the COVID-19 pandemic, more expertise, professional training workshops, and accredited training module(s) should be provided to social workers to back up the agency’s application in service delivery.*” (IFSC F)

“*Social workers could be more creative with ICT use and more flexible in exploring diverse service delivery modes in preventive activities, groups, programs, and casework. It was suggested that hybrid service delivery modes could be implemented in the family services in the future.*” (IFSC J)

“*As a professional in human service, a balance between online and in-person encounters needs to be made to provide a humane, dynamic and warm intervention to our service users.*” (IFSC G)

“*Using social media, enhancing intervention skills in an online programme, and using ICT tools were all new aspects and techniques different from traditional social work training. To adapt to the “new normal”, more evidence-based research about using ICT in social work intervention should be enhanced. Also, the cross-professional operation was necessary and unavoidable. Ethics in using ICT as intervention and providing social services should be the core study to set rules and regulations.*” (IFSC I)

“*Without direct face-to-face communication, some clients felt at ease to share their issues and overcome the feeling of embarrassment….For those topics which were sensitive and controversial like sexuality and social unrest, participants could feel more comfortable to raise questions by hiding themselves behind the cameras or writing their concerns in the chat room.*” (IFSC J)

## 4. Discussion

This paper is the first to systematically report the perceived ICT use in the family service and work process components (process evaluation) and their perceived benefits (outcome evaluation) amid the COVID-19 pandemic with quantitative and qualitative evaluation. The most common perceived increase in ICT use was group activities for people with specific service needs in the service component (54.5%) and activity promotion in work processes (48.6%). The top 3 most common perceived benefits of a perceived increase in ICT use were to provide another service option (74.2%), improve service convenience (60.2%), and maintain normal service (59.7%) under social distancing during COVID-19 outbreaks. Perceived benefits were significantly associated with perceived increased ICT use in different components. A clear picture of ICT use in family services and a better understanding of local service needs, social workers’ experience, lessons learnt, perceived barriers and preferences in ICT use amid the COVID-19 pandemic were reported. This study could help efficiently identify the key actionable strategies for enhancing ICT use in family and other services. 

The perceived increase in ICT use in different family service and work process components might relate to the increased demand for ICT-related services and the well-preparedness of our NGO partners to meet the unexpected challenges amid the COVID-19 pandemic. Since 2018, after the start of the JCSFL project, our NGO partners have been financially supported for hardware needs, including building a better IT infrastructure, enhancing internet connectivity, and purchasing mobile devices for frontline workers. Besides, our NGO partners’ respondents have been technically supported by the working team (SPH and TELI) of the JCSFL to meet software needs (such as face-to-face training sessions and a self-paced i-TLS online co-learning and sharing platform). These laid a good groundwork for ICT use and a relative readiness of NGO partners to face the service barriers related to IT use. They also provided an advantage for a smoother transition to ICT use in family services amid the pandemic. Based on the co-creation, co-sharing and co-learning model of the JCSFL project, our NGO partners were positively changed in their mindset, self-efficacy and skills of integrating ICT in family services, which echoed the positive factors leading to the ease of ICT use amid the pandemic [12]. The ICT use facilitated the incorporation and use of e-messages for activity promotion and implementation, using mobile applications (Apps) by service users to complete their group assignments, conducting interactive communication via instant messaging applications (such as WhatsApp and WeChat), and using video-conferencing software (such as Zoom) and other applications (such as Facebook Live Stream) for promotion and implementation. Similar changes in technology use and online practice were reported in studies on social work services [15,16,17,18]. Besides, our respondents found themselves more comfortable discussing sensitive topics online mode. They reported that attending online groups and programmes allowed them not to disclose their identities when seeking help, particularly on sensitive topics, such as sexual attitudes, lifestyles, and cultural-related issues. ICT use in discussing sensitive topics and collecting data on sensitive behaviours has been adopted to collect data on sensitive behaviours [19].

Regarding the perceived benefits of ICT use, more than half of the respondents reported perceived benefits of providing another service option, improving service convenience, and maintaining normal service, demonstrating the importance of ICT use when traditional face-to-face services had to be closed or restricted due to social distancing regulations for the control of COVID-19. Researchers have suggested the advantages of ICT use that enabled more client-driven service and attracted some user groups with special needs, e.g., busy parents and chronic illness caregivers [16]. Using online tools and platforms in various service referrals and applications can enhance timely applications and early resource availability for clients. Significant associations of the number of family service and work process components with a majority of perceived benefits, except improved service convenience and early identified high-risk groups, were reported by our social workers. These showed the limitations of ICT in some family services and work process components, especially for those involving more intensive counselling elements. In managing crisis cases, intensive face-to-face counselling contact is important to facilitate rapport building between service users and counsellors and should not be replaced by ICT. 

On the other hand, there were barriers to using ICT in frontline practice, such as challenges in observing non-verbal feedback and response while delivering online counselling services [16,20]. In addition, observing interactions among group members would be difficult in group work when the sessions are delivered on a virtual conference platform such as Zoom. Security issues in online frontline practice are major concerns that need further examination and tackling [7,8]. Nevertheless, the upsurge of suspected child abuse and domestic violence cases amid the COVID-19 pandemic requiring immediate tangible support, e.g., home/hospital visits and escort service, cannot be replaced by ICT. Another hurdle is the low ICT literacy of disadvantaged groups, limiting their use of ICT-assisted intervention, and similar findings have been reported elsewhere [21]. 

The strengths of the study included both qualitative and quantitative approaches to enrich the understanding of respondents’ feedback with the systematic and comprehensive outcome and process evaluation and using framework analysis to generate themes with organised structure (from service needs and social workers’ experiences, lessons learnt, perceived barriers and preference related to ICT use). The framework analysis originated in large-scale policy research. It offered valuable insight to inform implementation strategies [14] and has become increasingly popular in multi-disciplinary medical and health applied policy research to meet specific information needs with actionable outcomes [22]. Besides, this study provided evidence and a clear direction for further improvement in work processes, including activities planning and follow-up in family and other social services. 

Our study had several limitations. First, we measured perceptions and not actual ICT use. Perceived ICT use may not reflect actual usage and can influence an individual’s personality and self-perception [23]. Second, we used self-reported outcome-based questions to measure the perception of ICT use, and recall bias could not be ruled out. The perceived ICT use might be under or over-estimated depending upon different factors when completing the questionnaire. Third, the response rate was lower than expected, possibly because some workers were overwhelmed by the pandemic. Fourth, the generalizability of results might be limited, and non-adopters views were not available, even when we had included in the study all 26 NGO-operated IFSCs/ISCs out of the 67 IFSCs/ISCs (41 centres were operated by the government Social Welfare Department) in Hong Kong. Fifth, social desirability bias could result in over-reporting ICT use and benefits. Sixth, we did not collect qualitative feedback from the frontline social workers, which might help obtain additional information. Lastly, we could not affirm the causal relationship between the ICT use and perceived benefits as the study design was cross-sectional. 

From the feedback from social workers, we recommended some important elements to be considered for further enhancing ICT use in family services. First, ICT use in family services has benefited and is well-accepted by both frontline and managerial social workers. Second, hardware support, including IT infrastructure, network and equipment, is crucial to transforming traditional face-to-face contact into a new normal hybrid delivery mode. Third, continuous in-service training to enhance and update staff’s ICT-related knowledge and skills are needed to help adopt new technology and service development. Third, clear instructions and guidance on data security and management in ICT use are essential, which help build staff’s confidence and motivation to use different service components and broaden education. Fifth, ICT literacy is important for service providers (social workers) and users. Proactive support and training for subgroups such as the elderly are needed to enhance ICT literacy. 

## 5. Conclusions

To conclude, we have shown that perceived ICT use in family services increased amid the COVID-19 pandemic. Many perceived benefits were associated with the use in various service components and work processes. Our study has provided a comprehensive outcome and process evaluation of ICT use in service and work processes with quantitative and qualitative feedback and suggested actionable strategies to improve ICT use in service development. This paper should be a useful reference for researchers, health and social care professionals, and administrators for service evaluation and development. Further research on ICT use to promote and expand the social workers’ and related professionals’ services and work processes is warranted. 

## Figures and Tables

**Table 1:** The complexity of service needs, service targets, format and examples of four family service components.

Components	The Complexity of Service Needs	Service Targets	Format	Practice Examples
Casework ^1^	Moderate to high	Individuals and/or families with specific service needs	Brief and supportive casework for tangible service	Referrals for financial and residential placement and supportive counselling.
			Intensive counselling casework for complex family issues	Counselling sessions and multi-disciplinary case management for mental illness, suspected child abuse, domestic violence, tense interpersonal or marital relationships.
Groups ^2^	Low to moderate	Individuals sharing specific service needs, e.g., chronic illness carers’ stress	Four session group activities for at least six service users	Mutual support group, social skill training and volunteer training
Programmes ^3^	Low to moderate	A larger number of service users with some specific service needs	Activities with less than four sessions	Stress management workshops, psychoeducation and related activities to meet service users’ holistic well-being needs
Preventive family programmes ^4^	Low	General public to promote holistic well-being	Activities with less than four sessions	Psychoeducation and family well-being talks, social, recreational activities and community events

^1^ Casework = Brief supportive or intensive counselling for individuals and families with complex family issues and no limitation on the number of sessions. ^2^ Groups = Four session group activities for a specific group (at least six individuals) with special service needs. ^3^ Programmes = Activities with less than four sessions for many service users with specific service needs. ^4^ Preventive family programmes = Activities with less than four sessions for the general public to enhance holistic well-being.

**Table 2:** Characteristics of respondents (*n* = 255).

	*n* (%)
**Sex**	
Men	68 (26.8)
Women	186 (73.2)
**Age (years)**	
18–29	49 (19.4)
30–49	166 (65.9)
50–59	37 (13.9)
**Education**	
Undergraduate	115 (46.0)
Postgraduate	135 (54.0)
**Rank**	
Frontline social worker	228 (89.4)
Supervisor	27 (10.6)

Missing data were excluded.

**Table 3:** Changes in perceived ICT use in family service components during the COVID-19 pandemic.

	Decrease	No Change	Increase	Inapplicable/ No Use	Net Change *
	*n* (%)	*n* (%)	*n* (%)	*n* (%)	% Points
Casework ^1^	8 (3.1)	79 (31.1)	142 (55.9)	25 (9.8)	52.8
Groups ^2^	15 (5.9)	51 (20.0)	154 (60.4)	35 (13.7)	54.5
Programmes ^3^	17 (6.7)	71 (27.8)	106 (41.6)	61 (23.9)	34.9
Preventive family programmes ^4^	17 (6.7)	67 (26.4)	103 (40.6)	67 (26.4)	33.9

ICT: Information and communications technology. * Net change (percent points) = percentage increased minus percentage decreased. ^1^ Casework = Brief supportive or intensive counselling for individuals and families with complex family issues and no limitation on the number of sessions. ^2^ Groups = Four session group activities for a specific group (at least six individuals) with special service needs. ^3^ Programmes = Activities with less than four sessions for many service users with specific service needs. ^4^ Preventive family programmes = Activities with less than four sessions for the general public to enhance holistic well-being.

**Table 4:** Perceived ICT use in work processes before the COVID-19 pandemic and perceived increase in ICT use in work processes during the COVID-19 pandemic (*n* = 255).

	Perceived ICT Use before Pandemic		Perceived Increase in ICT Use during Pandemic	
	*n*	(%)	*n*	(%)
Planning ^1^	27	(10.6)	48	(18.8)
Promotion ^2^	102	(40.0)	124	(48.6)
Recruitment ^3^	75	(29.4)	98	(38.4)
Implementation ^4^	32	(12.5)	118	(46.3)
Evaluation ^5^	66	(25.9)	94	(36.9)
Follow-up ^6^	34	(13.3)	40	(15.7)
Others	16	(6.3)	34	(13.3)
Any process ^7^	149	(58.4)	208	(81.6)

ICT: Information and communications technology. ^1^ Planning refers to designing and preparing activities. ^2^ Promotion refers to publicizing the activities. ^3^ Recruitment refers to recruiting service users. ^4^ Implementation refers to conducting the activities. ^5^ Evaluation refers to collecting feedback from service users. ^6^ Follow-up refers to conducting follow-up services after activities. ^7^ Any process refers to the use of ICT in at least one component of the work processes.

**Table 5:** Perceived benefits and its association with the number of family service components with a perceived increase in ICT use during the COVID-19 pandemic (*n* = 221).

		Family Service Components		Work Process Components	
Perceived Benefits	*n* (%)	Adjusted Odds Ratio ^6,7^ (95% CI)	*p* Value	Adjusted Odds Ratio ^6,8^ (95% CI)	*p* Value
Provided another service option ^1^	164 (74.2)	1.38 (1.00, 1.90)	0.047	1.04 (0.83, 1.31)	0.74
Improved service convenience ^2^	133 (60.2)	1.25 (0.95, 1.65)	0.11	0.81 (0.66, 1.00)	0.054
Maintained normal service	132 (59.7)	1.29 (0.98, 1.69)	0.07	1.24 (1.01, 1.53)	0.041
Broadened service coverage ^3^	92 (41.6)	1.63 (1.23, 2.15)	0.001	1.41 (1.14, 1.74)	0.002
Reached the unreachable ^4^	58 (26.2)	1.19 (0.88, 1.60)	0.27	1.39 (1.08, 1.78)	0.009
Improved service efficiency	55 (24.9)	1.38, 1.02, 1.87)	0.03	0.92 (0.72, 1.16)	0.46
Early identified high-risk groups	10 (4.5)	1.55 (0.85, 2.80)	0.15	1.08 (0.64, 1.84)	0.78
Destigmatisation ^5,9^	2 (0.9)	-	-	-	-

ICT: Information and communications technology. ^1^ Provided another service option = Provided another (online service) option to service users. ^2^ Improved service convenience = Improved service convenience for service users to attend the activities. ^3^ Broadened service coverage = Broadened service coverage increased varieties and formats of activities. ^4^ Reached the unreachable = Connected with those who did not want or were unable to attend any face-to-face service. ^5^ Destigmatisation = not to be identified and marked as an undesirable characteristic in a way that narrows a person’s social identity. ^6^ Adjusted for sex, age, education and rank. ^7^ Adjusted odds ratios are for perceived benefit per family service component with increased ICT use (0–4). ^8^ Adjusted odds ratios are for perceived benefit per work process component with increased ICT use (0–4). ^9^ Adjusted Odds ratios for destigmatisation are not shown due to the small number.

## Data Availability

The data presented in this study are available on request from the corresponding authors.

## Supplementary Materials

The following supporting information can be downloaded at: https://www.mdpi.com/article/10.3390/ijerph192315634/s1, Table S1. The applications of i-Action co-created ICT tools and platforms in 4 family service components; Table S2. The applications of i-Action co-created ICT tools and platforms in the six work processes in family services; Figure S1. The examples of picture and animated e-messages on five strategic themes of family well-being promotion, and the 15 mobile game applications on three strategic themes; Figure S2. The screenshots of the E-message Portal and Evaluation Tool Builder

## References

[1] Cowling B.J., Ali S.T., Ng T.W.Y., Tsang T.K., Li J.C.M., Fong M.W., Liao Q., Kwan M.Y.W., Lee S.L., Chiu S.S. (2020). Impact assessment of non-pharmaceutical interventions against coronavirus disease 2019 and influenza in Hong Kong: An observational study. Lancet Public Health.

[2] Westbrook L. Alarming Rise in Hong Kong Young People Struggling with Mental Health Issues amid COVID-19 Pandemic, Experts Warn. South China Morning Post 17 October 2022. https://www.scmp.com/news/hong-kong/health-environment/article/3105943/hong-kong-witnesses-alarming-rise-young-people.

[3] Social Welfare Department Statistics on Child Protection, Battered Spouse and Sexual Violence Cases 6 May 2021. https://www.swd.gov.hk/vs/index_e.html#s3.

[4] Social Welfare Department Integrated Family Services 2005. https://www.swd.gov.hk/en/index/site_pubsvc/page_family/sub_listofserv/id_ifs/.

[5] Social Welfare Department Funding and Service Agreement. (n.d.). https://www.swd.gov.hk/doc/Subv_SAMPLE%20FSAs%202015/Family%20and%20Child%20Welfare%20Services/FS10-e.pdf.

[6] Department of Social Work & Social Administration, The University of Hong Kong (2012). Review of the Information Technology (IT) Strategy for the Social Welfare Sector—Final Report. https://www.swd.gov.hk/ngoitcorner/doc/en/it_strategy/FinalReportEnglish.pdf.

[7] Social Workers Registration Board Code of Practice. Social Workers Registration Board 15 January 2010. https://www.swrb.org.hk/en/Content.asp?Uid=14.

[8] Social Workers Registration Board Guidelines on Code of Practice. Social Workers Registration Board 15 January 2010. https://www.swrb.org.hk/en/Content.asp?Uid=15.

[9] Tsang W.H.W., Mo K.Y.H., Cheung J.C.S., Wong E.Y.W. (2022). Social Workers’ Acceptance of Information and Communication Technology (ICT) in Practice during COVID-19: Search for Embracing Ethical Considerations in Hong Kong. J. Soc. Serv. Res..

[10] Mishna F., Milne B., Sanders J., Greenblatt A. (2022). Social Work Practice during COVID-19: Client Needs and Boundary Challenges. Glob. Soc. Welf..

[11] Romea A.C., Valero D., Elboj C., Melgar P. (2022). Mental Health and Access to Information in Times of COVID-19: The Role of Social Work. Int. J. Environ. Res. Public Health.

[12] Suen M.M.T., Yuen A.Y.K., Wang M.P., Ho D.S.Y., Lam T.H. (2022). Development and Evaluation of an Innovative Web-Based Training, Learning, and Sharing Platform for Social Workers (Hong Kong Jockey Club SMART Family-Link Project): Mixed Methods Evaluation Study. JMIR Form. Res..

[13] Sit S.M.M., Lai A.Y.K., Kwok T.O., Wong H.W., Wong Y.L., Lam E.Y.W., Chan J.Y.W., Kong F.S.W., Cham K., Ng C.K.K. (2020). Process evaluation and experience sharing on utilising information communication technologies and digital games in a large community family health event: Hong Kong Jockey Club SMART Family-Link Project. Front. Public Health.

[14] Srivastava A., Thomson S.B. (2009). Framework Analysis: A Qualitative Methodology for Applied Research Note Policy Research. JOAAG.

[15] Mishna F., Fantus S., McInroy L.B. (2017). Informal use of information and communication technology: Adjunct to traditional face-to-face social work practice. Clin. Soc. Work. J..

[16] Mishna F., Milne E., Bogo M., Pereira L.F. (2020). Responding to COVID-19: New trends in social workers’ use of information and communication technology. Clin. Soc. Work. J..

[17] Razai M.S., Oakeshott P., Kankam H., Galea S., Stokes-Lampard H. (2020). Mitigating the psychological effects of social isolation during the COVID-19 pandemic. BMJ Br. Med. J..

[18] Wright J.H., Caudill R. (2020). Remote treatment delivery in response to the COVID-19 pandemic. Psychother. Psychosom..

[19] Burkill S., Copas A., Couper M.P., Clifton S., Prah P., Datta J., Conrad F., Wellings K., Johnson A.M., Erens B. (2016). Using the Web to Collect Data on Sensitive Behaviours: A Study Looking at Mode Effects on the British National Survey of Sexual Attitudes and Lifestyles. PLoS ONE.

[20] Hall J.A., Harrigan J.A., Rosenthal R. (1995). Nonverbal behavior in clinician–patient interaction. Appl. Prev. Psychol..

[21] Heponiemi T., Jormanainen V., Leemann L., Manderbacka K., Aalto A., Hyppönen H. (2020). Digital divide in perceived benefits of online health care and social welfare services: National cross-sectional survey study. J. Med. Internet Res..

[22] Gale N.K., Heath G., Cameron E., Rashid S., Redwood S. (2013). Using the framework method for the analysis of qualitative data in multi-disciplinary health research. BMC Med. Res. Methodol..

[23] John O.P., Robins R.W. (1994). Accuracy and bias in self-perception: Individual differences in self-enhancement and the role of narcissism. J. Personal. Soc. Psychol..

