# Supplementary material for: Use of Information and Communications Technology in Family Services and Its Perceived Benefits amid the COVID-19 Pandemic in Hong Kong—Jockey Club SMART Family-Link Project
Source: Int J Environ Res Public Health. 2022 Nov 24;19(23):15634. doi: 10.3390/ijerph192315634 (PMC9740009; doi:10.3390/ijerph192315634)

Supplementary information

Table S1. The applications of i-Action co-created ICT tools and platforms in 4 family service components.

	Functions	Service Components: Casework [a]	Groups [b]	Programmes [c]	Preventive family programmes [d]	Practice examples
**A. ICT Tools**						
1. E-messages	Graphic and animated e-messages developed on 5 strategic themes of family well-being promotion on SMART Emotion, SMART Coping, SMART Parenting, SMART Living Habit and SMART Communication#	✓	✓	✓	✓	E-messages with tips of effective parenting skills and were sent to parent participants after each parenting group session
2. Mobile game applications	Mobile game application developed on 3 strategic themes of family well-being promotion on SMART Emotion, SMART Coping and SMART Parenting#	✓	✓	✓	✓	Use of meditation app during group/programme session, and encouraged participants to extend this practice in their daily life<br><br>Possible scenario: A client used a mood diary app to jot down his/her mood change and discuss with social worker in the next counseling session
3. Digital family games	Larger scale ICT family games, e.g. augmented reality (AR), virtual reality (VR) and motion detection technologies			✓	✓	Possible scenario: Took a family photo requiring team challenges in an augmented reality setting
**B. ICT platform**						
1. E-message Portal	With a simple clip-art library and word editing functions, can disseminate e-messages in 2 forms, WhatsApp and Short Message Service (SMS)	✓	✓	✓	✓	Distributed effective parenting skills and reminders for the upcoming programmes
2. Evaluation Tool Builder	Build electronic forms, also support online polling and quiz	✓	✓	✓	✓	Conducted assessment using electronic forms before the counseling/group sessions, and organised online polls during the programme/group

a Casework = Brief supportive or intensive counselling for individuals and families with complex family issues and no limitation on the number of sessions.

b Groups = Four session group activities for a specific group (at least 6 individuals) with special service needs.

c Programmes = Activities with less than 4 sessions for a large number of service users with specific service needs.

d Preventive family programmes = Activities with less than 4 sessions for general public to enhance holistic well-being.

Table S2. The applications of i-Action co-created ICT tools and platforms in the 6 work processes in family services.

	Functions	Work processes						Practice examples
		Planning [a]	Promotion [b]	Recruitment [c]	Implementation [d]	Evaluation [e]	Follow-up [f]	
**A. ICT Tools**								
1. E-messages	Graphic and animated e-messages developed on 5 strategic themes of family well-being promotion on SMART Emotion, SMART Coping, SMART Parenting, SMART Living Habit and SMART Communication[#]		✓	✓	✓	✓	✓	Sent e-messages along with the IFSCs/ISCs newsletter to promote their upcoming events, created a tailor-made e-message using E-message Portal for recruitment, using e-message of specific theme(s) to programme /group participants to remind their learning of the session and or follow-up session as a booster.
2. Mobile game applications	Mobile game application developed on 3 strategic themes of family well-being promotion on SMART Emotion, SMART Coping and SMART Parenting[#]				✓	✓	✓	Played the games during the sessions, use the mood score as one of the indicators / sources of reference in evaluation and follow-up
3. Digital family games	Larger scale ICT family games, e.g. augmented reality (AR), virtual reality (VR) and motion detection technologies				✓			Played the games during the programme /group session to bring out the key message
**B. ICT platform**								
1. E-message Portal	With a simple clip-art library and word editing functions, can disseminate e-messages in 2 forms, WhatsApp and Short Message Service (SMS)	✓	✓	✓	✓	✓	✓	Created own e-messages and e-forms and use them for various work process. That included an e-form to explore users' preference for planning upcoming programmes /groups, construct specific mailing lists based on the market research and send out details of user-

2. Evaluation Tool Builder	Build electronic forms, also support online polling and quiz	✓	✓	✓	✓	✓	✓	preferred groups / programmes to specific target groups, build e-forms to support online registration, organise online poll during session to bring out the key message, an e-form for evaluation and follow-up to measure the impact of the groups / programmes

a Planning refers designing and preparing activities;
b Promotion refers publicizing the activities;
c Recruitment refers recruiting service users;
d Implementation refers conducting the activities;
e Evaluation refers to collecting feedback from service users;
f Follow-up refers to conducting follow-up services after activities;

Aims of SMART Emotion, SMART Coping and SMART Parenting, SMART Communication and SMART Living Habit

1. SMART Emotion: Leading to proper manifestation of positive emotional management
2. SMART Coping: Gaining resilience through the acquiring and practising effective coping skills
3. SMART Parenting: Promoting better parent-child relationship
4. SMART Living Habit: Promoting healthy living habit
5. SMART Communication: Promoting effective communication

Figure S1. The examples of picture and animated e-messages on 5 strategic themes of family well-being promotion, and the 15 mobile game applications on 3 strategic themes.

A. Examples of picture and animated e-messages on 5 strategic themes of family well-being promotion

**Theme**	**Picture e-messages**	**Picture e-message description**
SMART Emotion	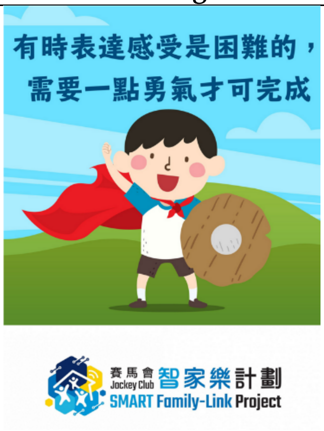	"Speak out your feeling is not easy sometimes, it takes you a little bit of courage to make it."
SMART Coping	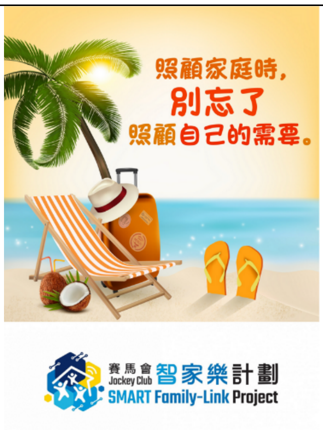	"When taking care of your family, don't forget your own needs."
SMART Parenting	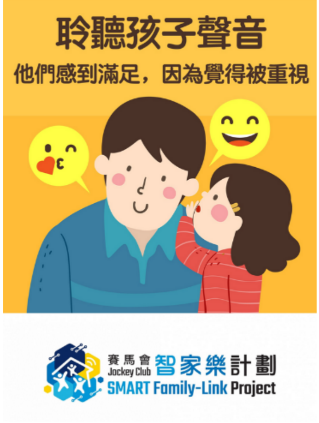	"Listen to your children. Let them feeling valued and be satisfied."
SMART Living Habit	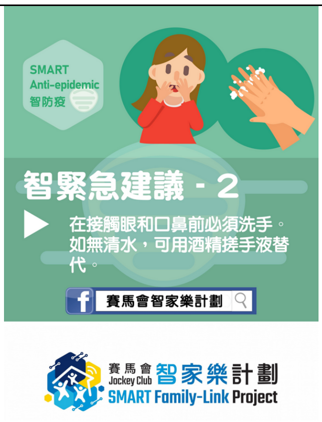	"Wash your hands before touching your eyes, mouth and nose. Use alcohol-based hand sanitiser if no soap and water are available."

Theme	Picture e-messages	Picture e-message description
SMART Communication	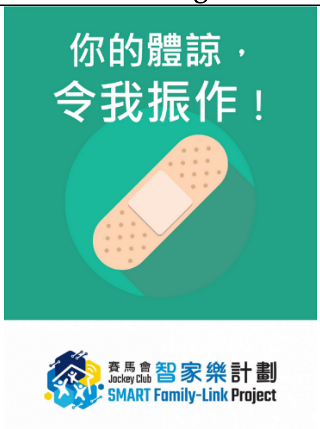	"Your understanding cheers me up."

B. Examples of mobile (game) applications (apps) on 3 strategic themes of family well-being promotion

Theme	Game applications	Description
SMART Emotion	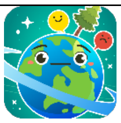 Emotion Kawaii	Build your own emotion icons and understand how to express your emotion.
	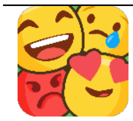 Emotion Kawaii ABC	Practise effective emotion management skills in 3 in-app mini-games.
	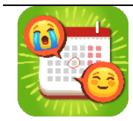 Emotion Kawaii D	Jot down everyday mood scores and events, and use the data visualization tool to monitor the changes
	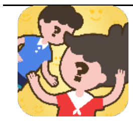 SMART Emotion – Phase 1	This is the first phase of a sequel of three. Based on cognitive behavioural therapy, the game series enables users to gain awareness and enhan emotional management by understanding th tripartite relationship between 'cognition 'behaviour' and 'emotion'.<br><br>In phase 1, users understand the association of 'emotion' and 'behaviour' through mini-games.
	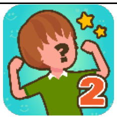 SMART Emotion – Phase 2	In phase 2, users understand associations o 'physiological symptoms', 'behaviours' and 'emotion' and build up their own profile. They ca gain further self-understanding objectively by compare their data with others.
	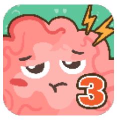 SMART Emotion – Phase 3	In phase 3, users will learn 'automatic thoughts', i.e. maladaptive thoughts leading to poor emotion management. They can then build up their 'thought diary' by extending their knowledge gained from phase 1 and 2 to gain a full understanding on the relationship among 'cognition', 'behaviour' and 'emotion'. Effective emotional management can be attained with proper identification of dominan automatic thoughts and application of effectiv thought stopping strategies.

Theme	Game applications	Description
SMART Coping	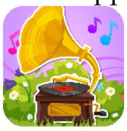 SMART Relax	Practise an effective coping strategy by practising audio-visual meditation.
	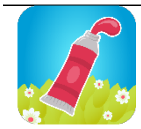 SMART Colouring Book	Practise an effective coping strategy by practising mindful drawing.
	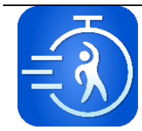 SMART Exercise	Practise physical exercises using AR technologies. Keep healthy body and positive mind.
	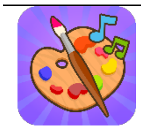 Mindstagram	Practise effective coping strategies by practising mindful drawing, audio-visual meditation in one-go. Personalised audio-visual meditation environment, online drawing exhibition platform are also available to users who want to achieve further.
SMART Parenting	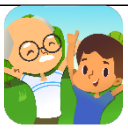 Follow Me	A simple memory game not only ask for the memory capacity, but also see how players collaborate in pairs.
	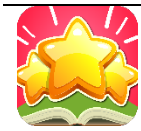 Star Stories 2	Reveal images of the stars and build your own story by linking them up! Challenge your friends or family members and win the game with the least time used! Increase the challenge by using your own photos and import them to the app.
	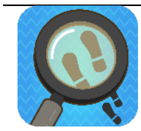 SMART Parenting – Phase 1	Enjoy a quality parent-child time on a family board game series! In phase 1, compete with your family members' knowledge on SMART Emotion, SMART Coping, SMART Parenting, SMART Communication and SMART Living Habit, win the game and build a personalised family photo using your unique profile picture!
	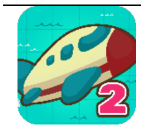 SMART Parenting – Phase 2	In phase 2, board game brings your family to travel around the world! Bring your family members around the globe and visit many hot destinations to compete the challenges. Win the game and build your own family photos using your unique profile picture and favourite destination.
	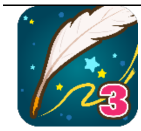 SMART Parenting – Phase 3	In phase 3, board game brings the family back and forth to visit our ancestors and to the future! Bring back the unique gifts from each important event of your parents by completing the challenges and win the game! Build your own family photos with the gifts and your unique profile picture.

Figure S2. The screenshots of the E-message Portal and Evaluation Tool Builder.

A. E-message Portal

a) Overview

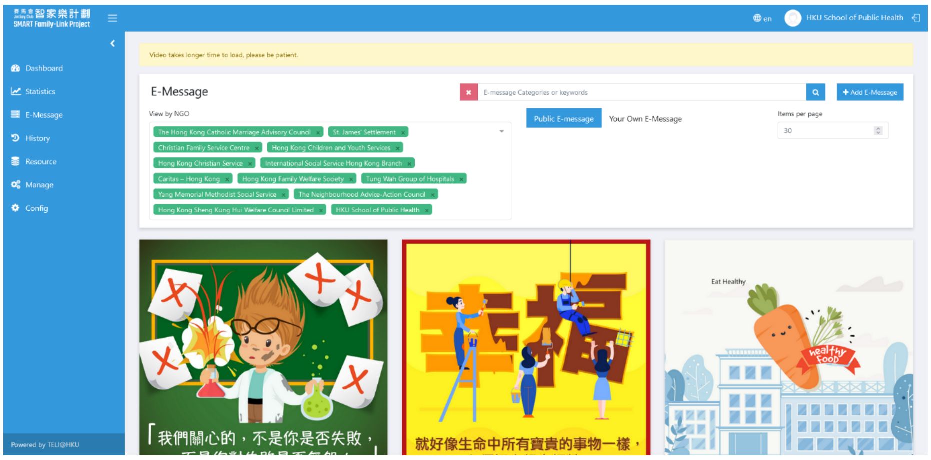

b) Built-in clip-art library and word editing functions

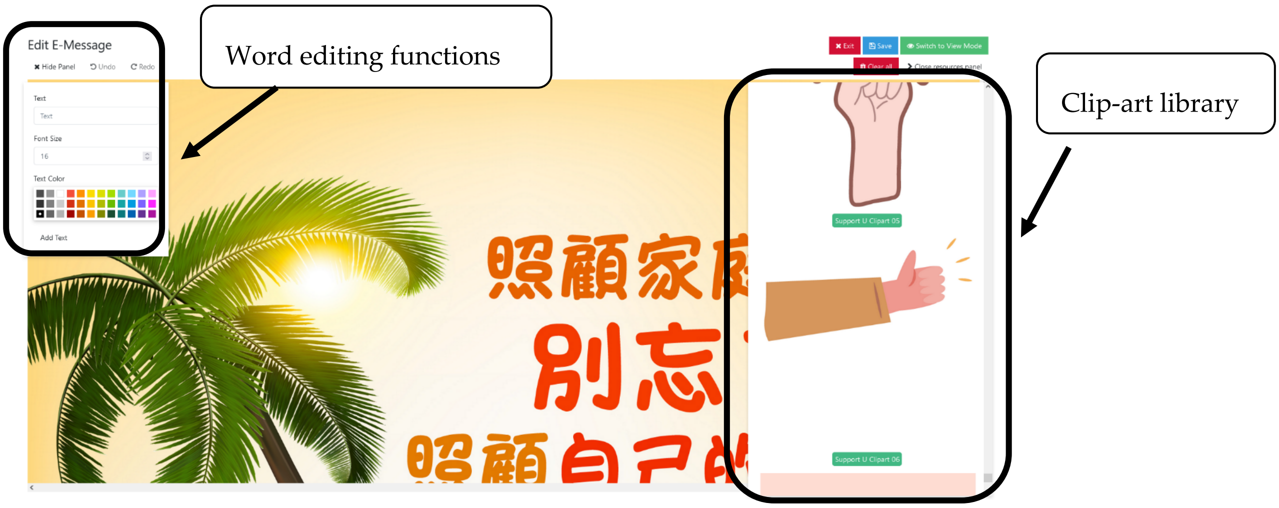

B. Evaluation Tool Builder

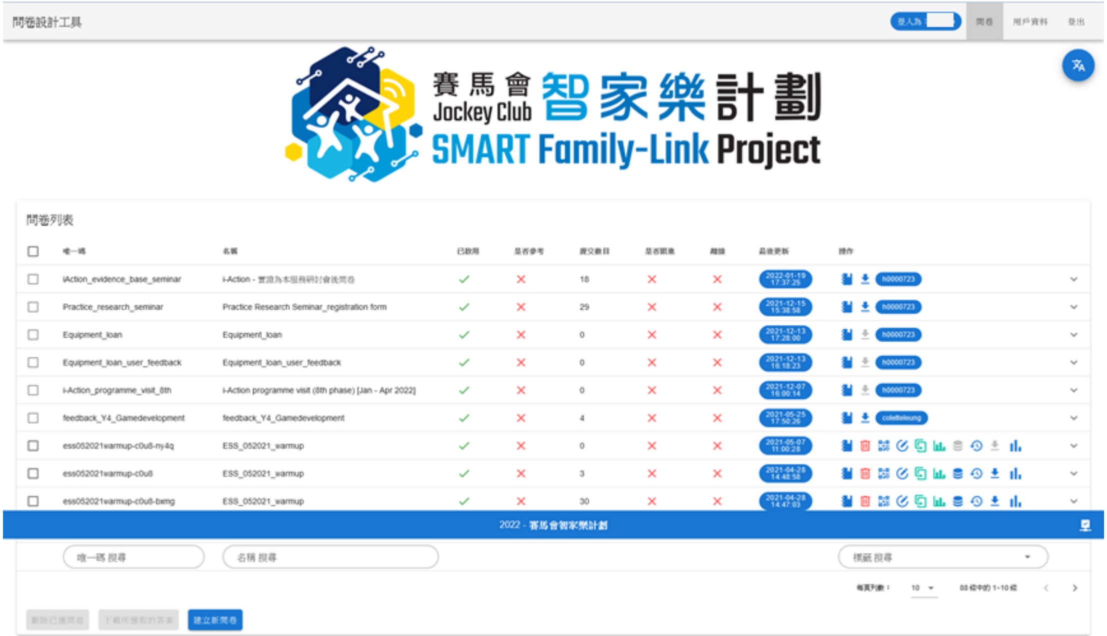

Supplement: Supplementary file 1 [file ijerph-19-15634-s001.zip › ijerph-1970416-supplementary.pdf]
